# Minimization of occurrence of retained surgical items using machine learning and deep learning techniques: a review

**DOI:** 10.1186/s13040-024-00367-z

**Published:** 2024-06-18

**Authors:** Mohammed Abo-Zahhad, Ahmed H. Abd El-Malek, Mohammed S. Sayed, Susan Njeri Gitau

**Affiliations:** 1 Department of Electronics and Communications Engineering, Egypt-Japan University of Science and Technology, New Borg El-Arab City, Alexandria Egypt; 2 Department of Electrical and Electronics Engineering, Assiut University, Assiut, Egypt; 3 Department of Electronics and Communications Engineering, Zagazig University, Zagazig, Egypt

**Keywords:** Retained surgical items, Artificial intelligence, Medical internet of things, Deep learning, Machine learning

## Abstract

Retained surgical items (RSIs) pose significant risks to patients and healthcare professionals, prompting extensive efforts to reduce their incidence. RSIs are objects inadvertently left within patients’ bodies after surgery, which can lead to severe consequences such as infections and death. The repercussions highlight the critical need to address this issue. Machine learning (ML) and deep learning (DL) have displayed considerable potential for enhancing the prevention of RSIs through heightened precision and decreased reliance on human involvement. ML techniques are finding an expanding number of applications in medicine, ranging from automated imaging analysis to diagnosis. DL has enabled substantial advances in the prediction capabilities of computers by combining the availability of massive volumes of data with extremely effective learning algorithms. This paper reviews and evaluates recently published articles on the application of ML and DL in RSIs prevention and diagnosis, stressing the need for a multi-layered approach that leverages each method’s strengths to mitigate RSI risks. It highlights the key findings, advantages, and limitations of the different techniques used. Extensive datasets for training ML and DL models could enhance RSI detection systems. This paper also discusses the various datasets used by researchers for training the models. In addition, future directions for improving these technologies for RSI diagnosis and prevention are considered. By merging ML and DL with current procedures, it is conceivable to substantially minimize RSIs, enhance patient safety, and elevate surgical care standards.

## Introduction

Retained surgical items (RSIs), objects inadvertently left within patients following surgical interventions, present a significant challenge within contemporary healthcare environments [1]. According to [2], approximately 0.3 to 1.0 cases of RSIs occur per 1,000 abdominal surgeries. Patients in such scenarios encounter grave repercussions, including infections, organ harm, extended hospitalization durations, and potentially fatal outcomes [3]. Additionally, these incidents expose healthcare providers to legal investigations and cast uncertainties on the standing of medical institutions [4]. Enhancing patient safety and diminishing the legal and reputational perils that healthcare practitioners and organizations encounter make the prevention of RSIs a pivotal concern in contemporary healthcare [5]. The healthcare sector is making strides toward significantly reducing RSI occurrences, augmenting patient well-being and healthcare standards. This objective can be realized by prioritizing monitoring, detection, and reduction strategies and harnessing the capabilities of integrated hardware-software solutions. Subsequent sections of this review article will analyze innovative methods and technologies employed for curtailing RSIs, shedding light on their merits, drawbacks, and potential for the future.

Three interlinked strategies have been adopted to tackle the issue of RSIs: monitoring, detection, and prevention. Monitoring involves tracking surgical instruments and materials throughout the procedure, ensuring accurate record-keeping at each phase [6]. Detection entails recognizing and localizing retained objects post-surgery, utilizing diverse imaging and identification methodologies [7]. Prevention is achievable through advanced methods to diminish the likelihood of items being left behind after the surgery [8]. Furthermore, patient safety and health are paramount; thus, there is a need to prevent RSI occurrences since they have physical, psychological, and emotional implications for patients.

Current RSI detection and prevention methods include manual counting, radiography, radiofrequency identification (RFID), and barcoding. These approaches largely rely on error-prone human accuracy and are time-consuming. Advanced technologies with comprehensive solutions encompassing hardware and software components are gaining momentum to address limitations in the current solutions [9]. Research on applying machine learning (ML) and deep learning (DL) technologies for RSI detection and prevention is ongoing [10, 11].

Due to the limitations of the currently used methods in minimizing RSI occurrences, ML and DL methods are preferred because of their higher accuracy in object detection [12]. These approaches offer numerous benefits, notably a substantial enhancement in the accuracy of RSI detection and the capacity to track surgical items in real-time during procedures. Their inherent ability to learn and adjust continuously enables them to steadily enhance their precision, diminishing the reliance on extensive human intervention and mitigating the potential for RSIs [13]. Ultimately, this elevates the quality of surgical care by streamlining surgical processes and enhancing patient safety.

Reviews relating to RSIs that have been done focus on conventional methods, analyzing their effectiveness and accuracy. This review explores cutting-edge advancements in healthcare. It investigates the possibilities for proactive and real-time monitoring to prevent RSIs by leveraging ML and DL. The study presents a forward-thinking viewpoint on a significant topic of patient safety. It offers new insights into how advanced technology can prevent surgical objects from being retained, providing more efficient and accurate solutions that align with the changing landscape of modern healthcare.

The paper is structured into eight sections. As described above, Section I introduces RSIs and their significance in healthcare. Section II is the methodology, detailing the approach adopted for article selection for review. Sections III and IV explore ML and DL techniques, expounding on their benefits and limitations. Section V discusses the primary datasets adopted in the different reviewed papers. Section VI is a discussion section that performs a comparative analysis of the technologies using results from the reviewed articles. Section VII examines the future directions in various technologies applicable to RSI occurrence minimization. Section VIII concludes the paper by summarizing key findings, stressing the importance of RSI prevention, and suggesting potential future healthcare advancements.

## Methodology

A comprehensive research strategy was developed to conduct a thorough literature review on minimizing retained surgical items using machine learning and deep learning. This strategy involved a systematic search across various scholarly databases, utilizing specific keywords to target relevant articles. The core keywords identified for this review were “retained surgical,” “machine learning,” and “deep learning.” These terms were selected based on their direct relevance to the research topic and frequent occurrence in existing literature.

**Fig. 1:**
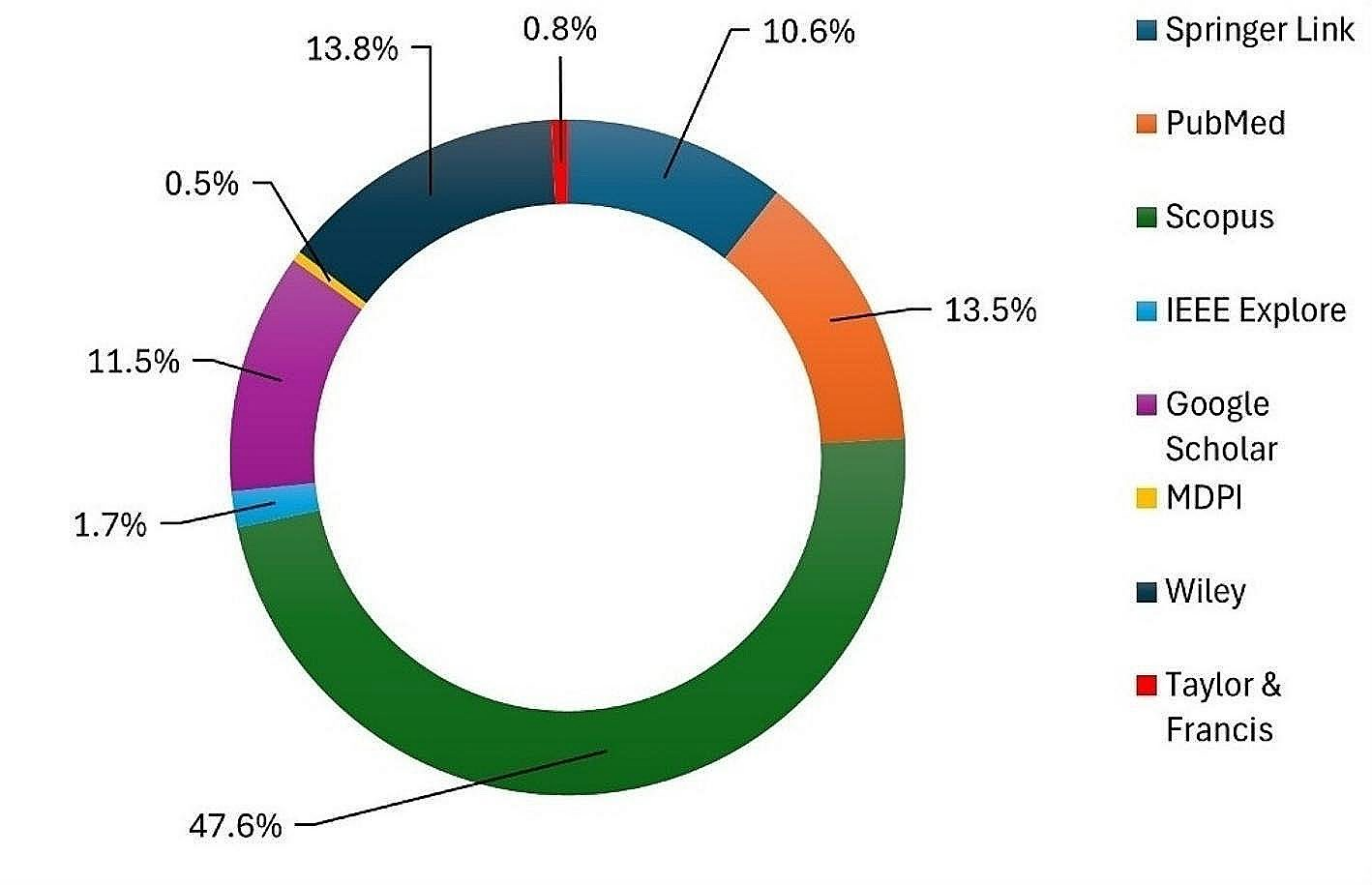
Distribution of the papers across publishers in step 1

**Fig. 2:**
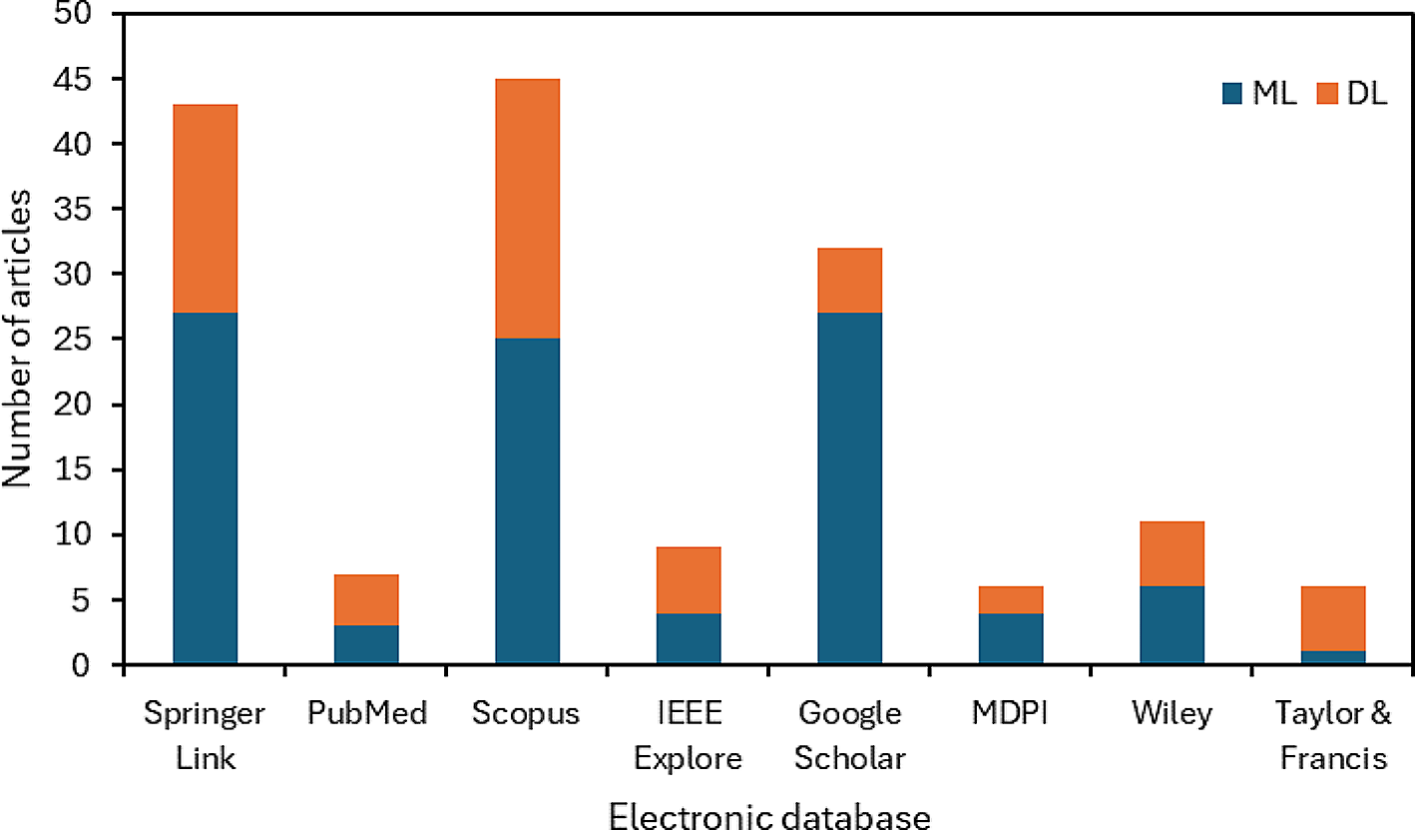
Results of step 2 for a more refined search

The databases selected for this search included Scopus, Springer Link, PubMed, IEEE Xplore, Google Scholar, MDPI, Wiley, and Taylor and Francis. These databases were chosen due to their extensive collections of medical and technological research articles. The search was initiated with the broad term “retained surgical” to capture many articles related to surgical items. This initial search yielded 4,236 results, including articles, books, reports, chapters, and special issues. The distribution of the results across the electronic databases is shown in Fig. 1. Two refined search queries were employed to narrow down the results by adding the phrases “machine learning” for ML-related papers and “deep learning” for DL-related papers. This second stage resulted in 97 ML articles and 62 DL articles; their distribution across the databases is shown in Fig. 2.

**Fig. 3:**
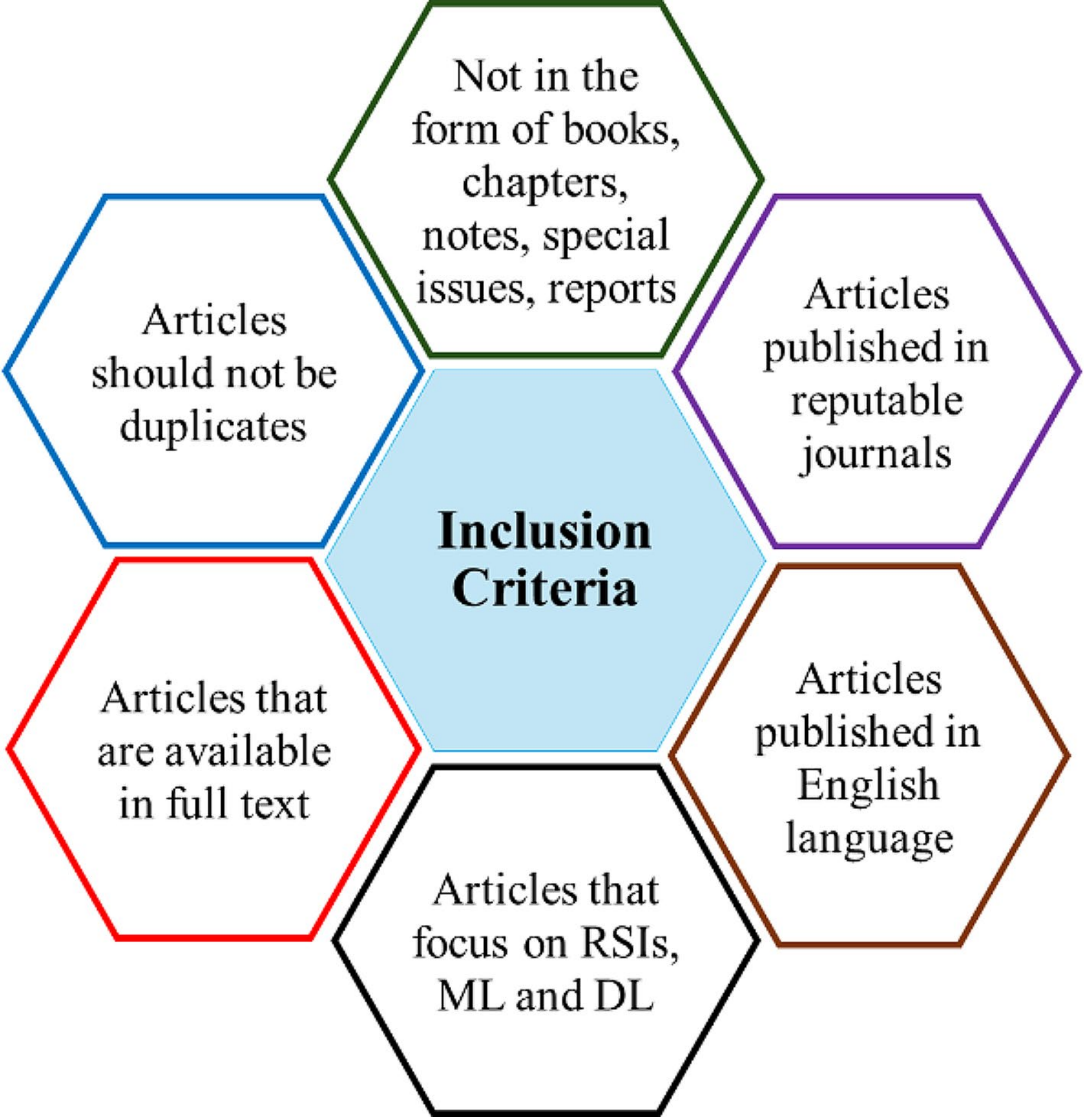
Inclusion criteria for reviewed articles

**Fig. 4:**
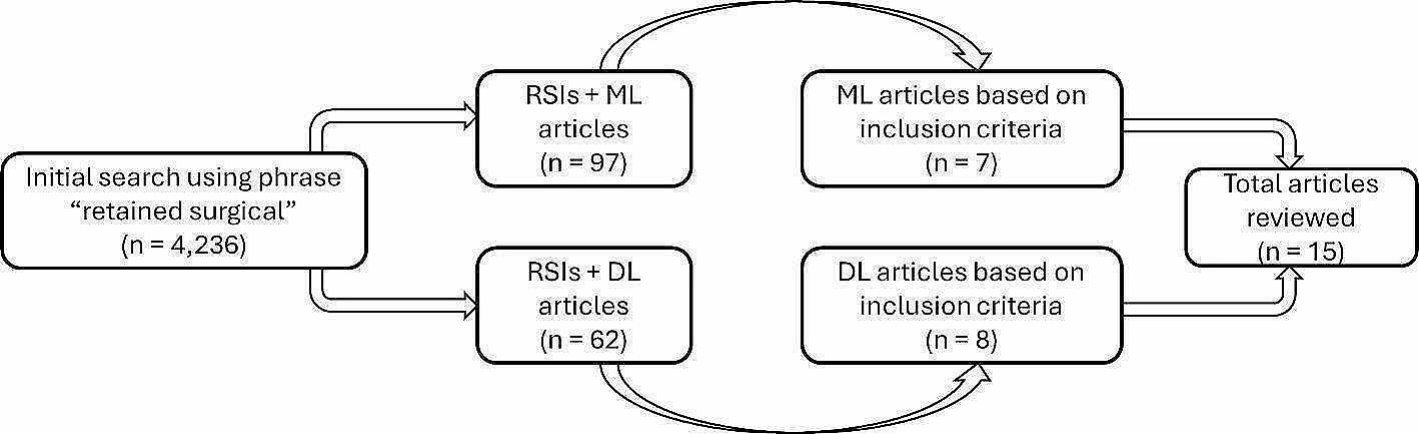
Stages of articles selection process

A third refinement step involving an inclusion criterion was applied to ensure that only the most relevant articles were considered in this review. The inclusion criteria adopted for this review paper are summarized in Fig. 3. Also, the articles were further scrutinized based on their titles, abstracts, and, where necessary, full-text reviews. This would enable the identification of the most pertinent studies, which would then undergo a quality assessment for final selection into the review. Quality assessment questions aim to assess the scope and address bias and validity. The five quality assessment questions used in this review are presented in Table 1. For each question, there are only three options for a response: Yes = 1; Partially = 0.5; and No = 0. Based on the responses to the questions, the quality of the articles was classified as either excellent (> 80%), very good (60 - <80%), good (40 - <60%), poor (20 - <40%), or very poor (< 20%) as indicated in Table 2. The articles with excellent, very good, and good scores based on the questions were selected, while those with very poor and poor scores were discarded. This research strategy was designed to ensure a comprehensive and systematic approach to gathering relevant literature, providing a robust foundation for the review article. A summary of the stages in selecting the 15 articles used in this review article is shown in Fig. 4. The integration of advanced technologies like ML and DL in this context is an emerging but increasingly notable area. However, the number of focused articles remains relatively small.

**Table 1:** Quality assessment questions

No	Item	Answer
Q1	Does the article describe clearly the study objectives?	Yes/Partially/No
Q2	Does the work provide adequate details on the research method?	Yes/Partially/No
Q3	Is there a description of the dataset characteristics and source?	Yes/Partially/No
Q4	Does the paper provide the architecture of the model used?	Yes/Partially/No
Q5	Did the article verify the results by relying on standard measures?	Yes/Partially/No

**Table 2:** Quality assessment scores

Quality scale	Very poor (< 20%)	Poor (20 - <40%)	Good (40 - <60%)	Very good (60 - <80%)	Excellent (> 80%)	Total
Number of articles	0	0	3	10	2	15
Percentage (%)			20	66.7	13.3	100

**Fig. 5:**
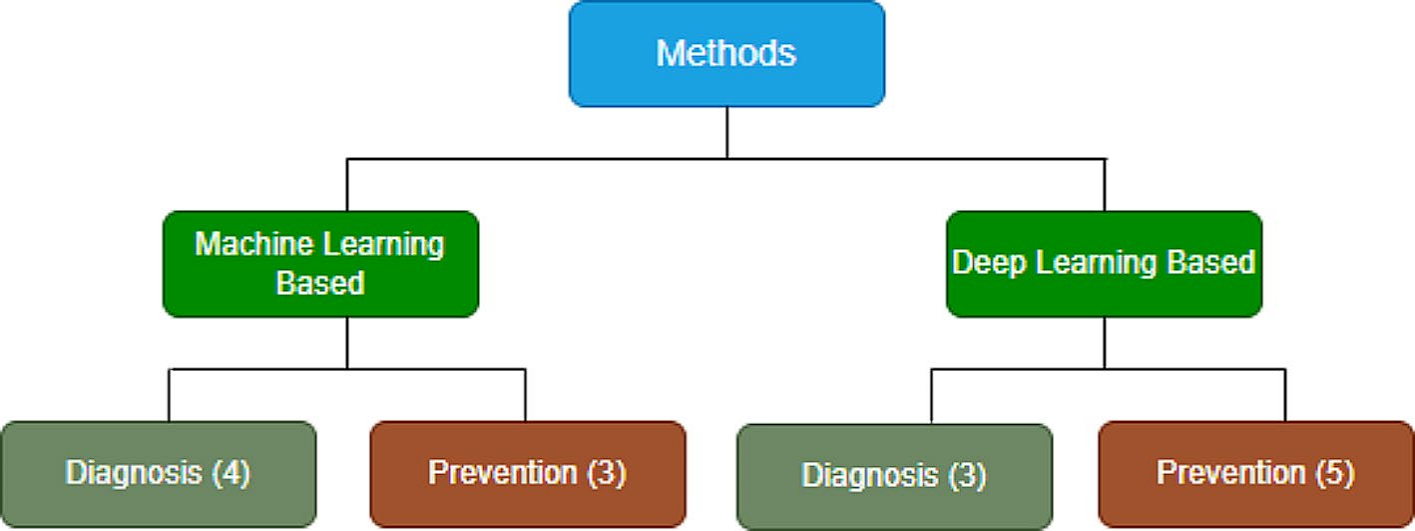
Review structure

The review follows a well-structured framework to comprehensively evaluate the application of ML and DL techniques in diagnosing and preventing RSIs. The reviewed articles are classified into two main categories, one for ML-based and the other for DL-based approaches. The categories are further subdivided based on the specific objectives, focusing on diagnosing and preventing RSIs. The first objective focuses on the diagnostic aspect, evaluating the role of ML and DL in enhancing the accuracy and efficiency of diagnosing RSIs. The second objective explores preventive measures and how these advanced computational techniques contribute to minimizing the risk of RSIs. This structure with the various classifications is shown in Fig. 5. This classification allows for a detailed examination of the methodologies employed within each category, offering a thorough analysis of the effectiveness of ML and DL techniques in addressing the problem. It provides a clear and insightful overview of the current state of research in the field, thus highlighting the advancements, challenges, and potential future directions for utilizing ML and DL in enhancing surgical safety.

## Machine learning-based methods

For decades, ML has transformed a wide range of industries by training computers to make predictions based on existing data. Much effort has been put into enhancing ML accuracy, and continual efforts are being made to incorporate these technologies into real-life applications [14]. ML has quickly moved beyond its early digital uses, such as email filtering, to other areas, such as healthcare. Aside from image recognition, language processing, and data mining, ML techniques are finding an expanding number of applications in medicine, ranging from automated imaging analysis to diagnosis [15]. Several researchers are looking into ML as a potential solution to improve medical care and surgical procedure safety [16]. Advanced technologies that use ML algorithms to track a surgical process in real time and send out appropriate notifications may reduce the incidence of RSIs.

### Machine learning models

Several ML models have already been studied and tested to have the capability to detect and track RSIs. The Random Forest model comprises several decision trees to enhance prediction accuracy and foster control over overfitting. Random forests do this by learning from diverse subsets of the data; they can smooth out possible overfittings in decision tree models, and by averaging predictions, it is possible to obtain a robust solution even from noisy data sources [10]. Featured in multiple studies [10, 17, 18], the model was utilized to improve predicting accuracy in RSI detection processes.

Neural networks, especially weighed multilayer perceptrons, are employed to classify the possible instances of RSI from radiologic images. This type of neural network is composed of several layers of neurons with non-linear activation functions, which makes the model able to learn unbelievably complex patterns or relationships in input data [18]. Multilayer perceptron can manage the problems of classification of high-dimensional data and detailed image classifications where a precise feature analysis is needed to get better results. Linear discriminant analysis (LDA) seeks a combination of features that would constitute a linear way of separating two or more categories of objects or events. Several studies have succeeded in using LDA to statistically strategize image features to discover RSIs’ presence [18]. The application of statistical methods for analyzing different types of surgical materials based on their radiographic appearances assists in distinguishing between surgical tools.

Instead of the traditional convolutional networks designed for image labeling, which aim to classify in general terms, the fully convolutional networks (FCNs) are designed to precisely label each pixel, which is essential when pinpointing the localization. The researchers of the FCNs have participated in segmenting surgical instruments of a non-rigid nature from video feeds [19]. Through the modification of convolutional layers from fully connected layers, FCNs consequently preserve the spatial designation, leading to the highly accurate pixel-level segmentation of objects. Along with these FCNs, optical flow tracking works best for real-time tracking of surgical tools. It calculates the object’s motion from one frame to the consecutive frame based on the visible changes [19]. This method is of fundamental importance to preserving the continuum of segmentation in video sequences, especially when containers drift or deform between frames.

### Diagnosis

**Fig. 6:**
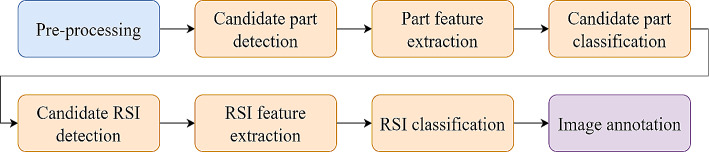
Computer vision and ML approach for detection of RSIs

The process of detecting RSIs by physician analysis of X-ray images is relatively lengthy, taking approximately 45 min to complete, and is susceptible to errors since it relies on the sharpness of the human eye for RSI detection. In [17], the researchers developed computer-aided detection (CAD) software to analyze X-ray (XR) images. By utilizing the large image data currently available, a system was proposed based on computer vision and ML algorithms, as shown in Fig. 6. Test images were obtained by placing 2–3 sponges on either a turkey or phantom model and using actual XR images that had clutter but did not have any sponges, generating a total of 790 images of which 277 had sponges. The software may be implemented as a stand-alone application, be part of a picture archiving and communication system (PACS), or be used in portable XR machine software. The software was effective based on the preliminary experiments conducted using actual XR images. The obtained results had 90% sensitivity, 99% specificity, 95% precision, 92% F-measure, and 99% accuracy.

Interpretation of radiographs by radiologists is a tedious process and heavily relies on human accuracy. The integration of ML and CAD holds potential for analyzing radiographs. In [20], the main aim was to assess the precision of a CAD technique in recognizing instances of RSIs. This was achieved by utilizing a distinct radiopaque tag that doesn’t deform. The approach involved building a CAD system using separate sets of radiographs for training and validation. A fresh collection of radiographs from cadavers containing both tagged and non-tagged artificial objects was then processed through the CAD system. Radiologists subsequently evaluated the CAD system’s negative classifications. The findings indicated that in collaboration with a failsafe radiologist, the CAD system achieved a solid ability to accurately detect the non-deformable radiopaque tag, displaying high levels of sensitivity and specificity. This combined approach facilitated swift and precise identification of the tag’s presence.

The adverse health effects and deaths related to the retention of foreign objects after surgery are substantial. According to [21], these incidents contribute to unnecessary medical expenses of $1.5 billion annually. Radiography has a limited success rate of 59% in detecting such retained objects. To tackle this issue, the authors employ two complementary technologies: a three-dimensional (3D) Gossypiboma Micro Tag (referred to as “Tag”), which enhances the visibility of these objects in radiographs, and a CAD system that identifies the presence of the Tag. The Tag’s 3D structure allows it to be effectively identified by radiologists, and the CAD system generates a consistent 2D image on radiographs, regardless of the object’s orientation within the body. By arranging the “Tag” and various common artificial objects in random patterns, a database of cadaver radiographs was established. CAD modules are developed for preprocessing and tag enhancement. The CAD system can function with high specificity for surgeons, fitting seamlessly into their workflow and serving as an initial evaluator. For precise detection, radiologists can employ the CAD in a high-sensitivity mode. In an evaluation involving 346 cadaveric radiographs, the CAD system demonstrated robust specificity for operating rooms (85.5% sensitivity, 0.02 false positives per image) and high sensitivity for radiologists (96% sensitivity, 0.73 false positives per image).

#### Top of form

Aaccidental retention of surgical tools within patients’ post-surgery can result in severe consequences. To address this issue, the research in [18] proposes using CAD on postoperative radiographs as a preventive measure. This CAD system could serve as an additional check for surgeons and radiologists, aiding patient safety during surgeries. The study aims to identify surgical needles in radiographs, developing a CAD system with the necessary sensitivity and specificity for recognizing these objects.

The CAD solution employs techniques like image segmentation, enhancement, feature analysis, and curve fitting to identify surgical needles in radiographs [18]. In [18], the dataset was compiled, including both “normal” cadaver images with and without needles. A reference standard was established using a graphical interface to locate needle positions precisely. The dataset was split into training and test sets, each accommodating two operational modes for the CAD system – one prioritizing specificity and the other emphasizing sensitivity. Results indicated that the rule-based classifier in the CAD system achieved sensitivities of 89.8% with 0.36 false positives per image in sensitivity-focused mode and 74.6% with 0.15 false positives per image in specificity-focused mode for the training set. For the test set, the CAD system demonstrated 77.2% sensitivity with 0.26 false positives per image in specificity-focused mode and 88.1% sensitivity with 0.28 false positives per image in sensitivity-focused mode when utilizing the neural network classifier. Overall, this novel CAD system effectively identified retained surgical needles in radiographs, presenting a promising solution to detect accidental retention within patients’ bodies at an affordable cost top of form.

### Prevention

**Fig. 7:**
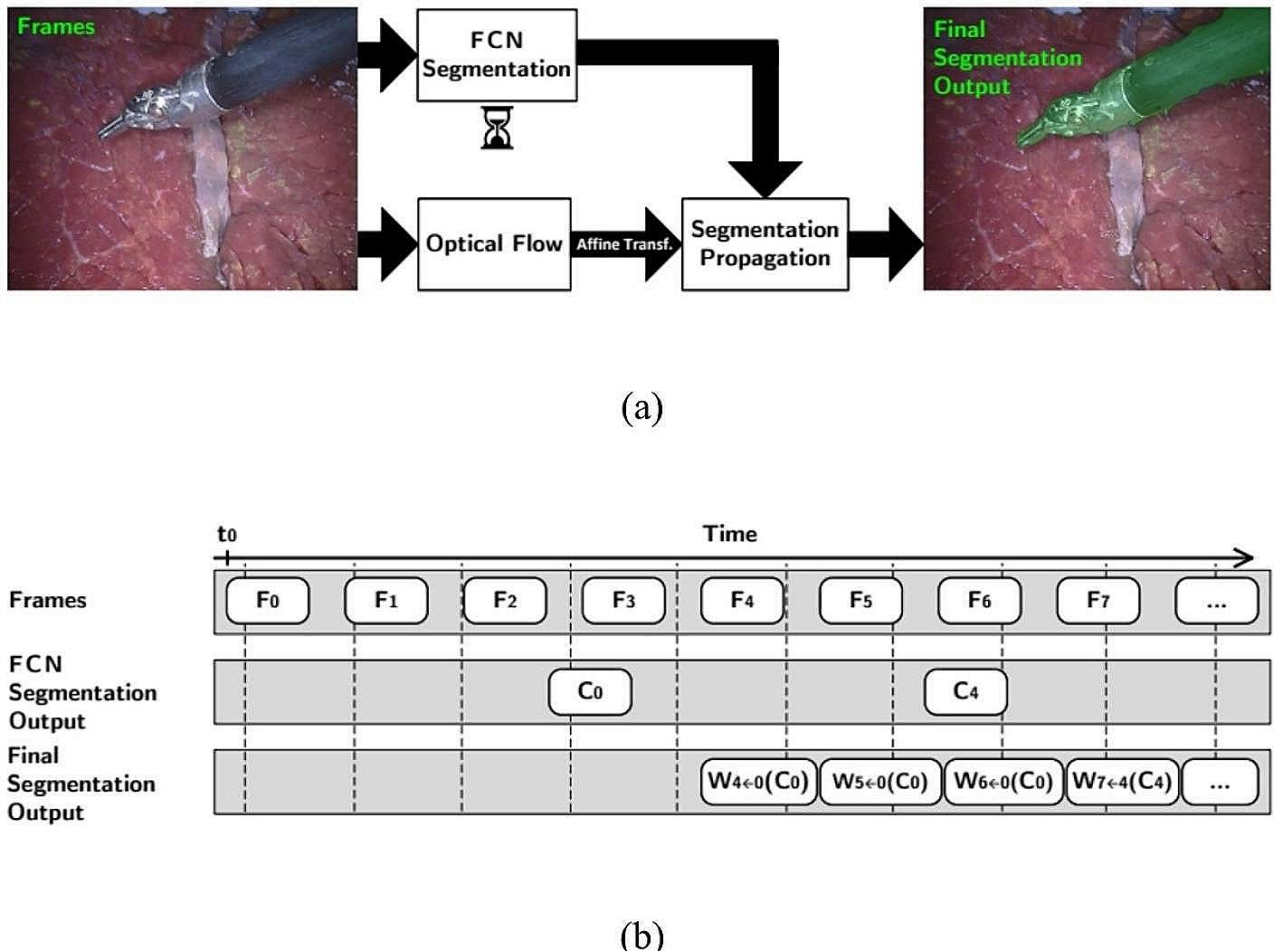
An automatic real-time image segmentation based on optical flow tracking and FCN. (**a**) A system proposed in [19], (**b**) Real-time diagram

In [19], the authors explained the significance of real-time segmentation in computer-aided systems used in surgical procedures. As illustrated in Fig. 7, they proposed an automatic approach based on optical flow tracking and FCNs. Initially, no segmentation from FCN is present, leading to no system output. Once the initial FCN output is acquired, continuous per-frame segmentation occurs. All segmentation results are derived from the latest FCN-based creation.

Deep neural networks and high-speed optical flow are used to produce accurate segmentation. The study used existing and new datasets from both in vitro and in vivo clinical cases for validation. The non-real-time method achieved an 89.6% accuracy, which performed better than the real-time method (combining DL with optical flow tracking) by 3.8%. The average balanced accuracy for the real-time method was 78.2% in the validated datasets.

Several factors contribute to “never events” (retained foreign items and wrong-site surgery) in operating theatre rooms [22]. According to [10], little information is known about the quantified risks of major “never events” and the characteristics of different surgeries. The authors employed ML principles to identify and quantify these risk factors to improve patient outcomes. To identify risk factors contributing to “never events,” 9,234 safety standards observations, 101 actual “never events,” and three ML models were used. The metrics of the three models were evaluated using a 10-cross validation technique to measure their impact on two “never events.” In 6 surgical departments, 24 contributing factors were identified: 6 with an impact of > 900%, 6 with 0–900% impact, and 17 with an impact of < 0%. The paper proposed adjusting safety standards based on risk assessment of the surgery and the surgery room. The study focused on just six surgical departments and was based on analysis of actual “never events”; thus, it may be biased and may not represent all surgical errors.

The overall key findings from various studies highlight that ML methods have achieved high accuracy and precision in detecting surgical items and instruments in medical images. Some methods indicate significant efficiency improvements, potentially reducing the time needed to perform surgical count and analysis. The main advantages of implementing ML in surgical count include enhanced detection capabilities that could increase patient safety by identifying retained surgical items (RSIs). By automating the count procedure, these systems also help reduce human analysis errors. Additionally, several of these ML methods can be seamlessly integrated with existing medical systems like the picture archiving and communication system (PACS), making them a versatile addition to current medical infrastructures.

Some of the drawbacks of the performed studies include limited datasets, which impacts the generalizability of the findings. The effectiveness of these ML systems also heavily depends on the quality of the input data, such as image quality and the specific characteristics of the RSIs. While applications of ML methods in surgical count show promising potential for improving patient outcomes and operational efficiency, their practical application remains constrained by issues related to data and system limitations. Continued research and the development of more diverse and extensive datasets are essential for advancing these technologies.

Table 3 includes the key findings and the main advantages and disadvantages of the ML methods described in [10] and [17–21].

**Table 3:** Machine learning methods key findings, prominent advantages, and disadvantages

Modality (Dataset)	Metrics	Key findings	Advantages	Disadvantages
X-Ray radiography (790 images) [17], 2015	• Specificity: 99% • Sensitivity: 90% • Precision: 95% • Accuracy: 99% • F-measure: 92%	• Experimental results show the effectiveness of the proposed approach. • 10-fold cross-validation on 790 XR images (277 with sponges). • A total of 561 sponges in the test collection. • Preliminary data confirm feasibility, suggest expanding the detection set, and prepare for further testing.	• The proposed CAD software enhances patient safety through RSI detection in XR images. • Computer vision and ML reduce human analysis errors. • Experiments show high specificity, sensitivity, precision, accuracy, and F-measure. • Integration options: PACS, stand-alone software, portable XR machine software.	• A limited dataset requires larger-scale testing for validation. • Superimposition process quality affects the accuracy of the proposed approach. • Tested only for detecting sponges; more research is needed for other RSIs. • Effectiveness can vary based on RSI appearance, location, and orientation. • Manual analysis by physicians is still essential for comprehensive detection.
Observations [10], 2023	-	• In 6 surgical departments, 24 contributing factors were identified. • 15–20 pairs with a higher probability of occurrence in 5 departments. • Three random forest models were employed, showing strong performance (AUC 0.81–0.85).	• ML identifies hidden patterns and offers better insights. • The random forest model tackles complexity and is accurate. • Non-binary features are simplified through discretization.	• The study’s hospital-focused data limits the findings’ generalizability. • Only two “Never Event” types were considered, not others. • Patient-related factors are not factored in. • Human, environmental, and organizational factors are not considered.
X-ray radiography (208 cadaver images) [18], 2017	-	• CAD system developed for automated surgical needle detection post-surgery. • CAD system effective in high sensitivity, high specificity modes. • Potential as low-cost aid in reducing retained surgical needles.	• CAD enhances needle detection accuracy in X-rays. • CAD performs well in high sensitivity, high specificity modes. • Classifier comparison reveals sensitivity vs. false positive trade-offs.	• The study’s small dataset covers only two needle types and lacks scenario diversity. • The dataset uses cadaver radiographs, not fully replicating real-world conditions, limiting the findings’ generalizability. • The CAD system’s sensitivity and specificity are relatively low, suggesting improvement with the larger dataset is needed.
X-ray radiography (700 thoracoabdominal images) [20], 2018	**CAD**: • Sensitivity: 79.5% • Specificity: 99.7%. **Radiologists**: • Sensitivity: 92.9–100% • Specificity: 99.3–100%. **CAD + 1** • Sensitivity: 98.5–100% • Specificity: 99.0-99.7%.	• Radiologist agreement is high, nearly perfect pairwise.	• CAD enhances accuracy, high sensitivity, and specificity. • CAD’s automation, with no specialized training, is a convenient, efficient tool. • CAD analyzes < 1 min/radiograph, offers speedy results, and process streamlining.	• The small sample size (9 cadavers) limits the findings’ generalizability. • The study didn’t test the high-sensitivity CAD + radiologist combo. • The CAD system was not validated on the separate test set, affecting generalizability. • There is no comparison with other methods, and relative strengths/weaknesses are missing. • There is no data on the CAD system’s impact on clinical outcomes or cost savings.
X-ray radiography (346 cadaver images) [21], 2014)	• Sensitivity: 96% • Specificity: 85.5%	• The paper suggests 3D µTag, CAD for RFOs: detection enhancement.	• Reliable detection: µTag’s 3D shape ensures accurate detection by radiologists and CAD. • CAD performance: High specificity for OR, high sensitivity for radiologists. • Workflow integration: CAD can seamlessly aid surgeons as the first reader.	• Limited dataset: The study uses 346 cadaveric radiographs, not fully real-world. • Lack of specific criteria info for CAD labelling, segmentation, analysis, and classification. • There is no mention of limitations for real-world implementation. • Cost-effectiveness and feasibility details for technology adoption not elaborated. • There is no info on the CAD system’s long-term impact on reducing retained objects in practice.
Optical imaging (EndoVisSub: 4760 images NeuroSurgicalTools: 2476 images FetalFlexTool: 21 images + a video sequence) [19], 2017	**EndoVisSub** • Sensitivity: 72.2% • Specificity: 95.2% • Accuracy: 83.7% **NeuroSurgicalTools** • Sensitivity: 82.0% • Specificity: 97.2% • Accuracy: 89.6% **FetalFlexTool** • Sensitivity: 84.6% Specificity: 99.9%Accuracy: 92.3%	• Validation using existing and new benchmark datasets. • Ex vivo and in vivo clinical cases with various surgical instruments. • There are two method versions: non-real-time and real-time. • The real-time version combines DL with optical flow tracking.	• The method is efficient and less time-consuming. • Accurately segments deformable parts of instruments in real-time. • Useful for instrument localization and separation from tissue. • The technology accurately segments extremely deformable surgical tools in real-time using optical flow tracking and FCN. • The ability to detect various instruments and the presence of a framework for tracking and learning detection.	• Quality of results affected by instrument deformability and movement speed. • FCN in a proposed method, not real-time, impacting surgical scenarios. • Challenges in real-time detection and tracking of surgical instruments include specular reflections, changing lighting conditions, motion blur, and occlusions caused by body fluids and smoke. • Challenges faced during the fine-tuning process and real-time instrument segmentation in surgical scenarios

## Deep learning-based methods

DL, a subset of ML, is transforming a variety of industries. This component employs multilayer artificial neural networks to process complex data structures [23]. The analysis and use of DL in numerous applications has yielded excellent results. DL has enabled substantial advances in the prediction capabilities of computers by combining the availability of massive volumes of data with extremely effective learning algorithms. Image recognition, object detection, self-driving cars, medication research, and disease diagnosis are just a few of the complex applications that have benefited from this advancement in ML [24]. Further research into the topic is critical since it may provide significant discoveries with practical applications. While traditional ML requires supervised feature extraction, DL can independently discover and evaluate non-structural input objects such as images and audio recordings [25]. After the lower layers have processed the input data or learned the simple features, the upper levels pick up on the complex features. DL is transforming healthcare, particularly in predictive analytics and diagnostic imaging [26]. One application is to improve the safety and precision of surgical procedures. This method could lead to better detection and warning systems for use in real-time surgery. Not only does it reduce the risk of surgical errors, but it also allows for a more intelligent surgical environment, which helps both surgeons and patients.

### Deep learning models

With the aid of technology, many researchers are working on deep learning techniques suitable for image detection and classification [27]. Owing to their capability of extracting image features at different levels of hierarchy, convolutional neural networks (CNNs) are crucial to image processing tasks [28]. One of the strengths of this model is that it is very useful for applications that require real-time tracking; thereby, it can be used for surgical tool detection during operations [29]. Spatial transformer networks (STNs) combine with convolutional neural networks to make it possible to adjust spatial data. This attribute plays a vital role when the objects of interest are constantly moving, such as when the surgical tools are displaced from their initial placement [28]. STNs consider these disturbances while transforming feature maps into a canonical and analyzable representation.

The U-Net structure stands out from others and is dedicated to segmentation tasks. The contracting path keeps the details in context, and the expanding symmetric way quickly acquires location [27]. This is also critical in medical image segmentation, where one needs to outline and define objects, such as RSIs. Faster region-based convolutional neural networks (R-CNN) gained wide appreciation as they were the first to reliably detect objects due to the use of region proposal networks (RPN) and the Fast R-CNN component [30]. This model has the potential to achieve higher degrees of sensitivity and specificity, accurately identifying surgical items in images, which is paramount for medical diagnosis.

### Diagnosis

Despite proposing a novel DL software that uses post-processed images created by combining X-ray images of typical post-operative radiography and surgical sponges, researchers have not fully investigated the relationship between the detectability of RSIs and human visual assessment. In [30], a study examined the relationship between the detectability of RSIs and human subjectivity using DL. A DL model was created using 2987 training shots and 1298 validation images that were post-processed by integrating X-ray images of typical post-operative radiography and surgical sponges. A second batch of 800 pictures was also used, 400 with and 400 without surgical sponges. The researchers used receiver operator characteristics to see how well a DL network and a general observer with ten years of clinical experience spotted retained sponges. The radiologist and the DL model determined the following values: The areas under the curves were 0.87 and 0.76, the cutoff values for probability were 0.37 and 0.45, and the sensitivity and specificity were 85% and 61%, respectively. When detecting surgical sponges, the DL model had higher sensitivity, while the human observer had higher specificity. These attributes suggested that the DL system, combined with human intervention, could improve clinical processes in operating rooms and effectively detect RSI.

When objects are accidentally left inside the brain during neurosurgical procedures, they can create potentially deadly health problems and necessitate invasive reoperations. A typical example of such retained surgical instruments is the cotton ball, which absorbs blood for improved surgical vision but becomes optically indistinguishable from brain tissue. However, due to their differing acoustic qualities, ultrasonic imaging can identify the difference between brain tissue and cotton. In [31], a fully automated approach for locating foreign bodies was developed. This algorithm was quickly integrated into the clinical workflow to discover and identify trapped cotton balls inside the brain. The DL method employed CNN, yielding 99% accuracy, sensitivity, and specificity. It outperformed comparable algorithms in the process. The technique was also turned into valuable applications like web and mobile user interfaces. The approach could detect a single cotton ball in an ultrasound image in less than a second. This study was notable for using a foreign body recognition algorithm based on actual in-person datasets for the first time. The findings indicate its usefulness in preventing unintended foreign body retention in a translational healthcare setting.

According to [32], 70% of RSIs are surgical sponges as they are more challenging to detect than other surgical items. The authors developed a CAD software program specialized in the diagnosis of retained surgical sponges. The program employed DL techniques to detect RSIs easily and effectively with high specificity and sensitivity. Using training and validation datasets, the software was developed by training it through DL. The dataset had 4554 training subjects and 470 validation subjects, created by the fusion of normal postoperative radiographs with surgical sponge radiographs. Phantom radiographs were taken using cadavers with surgical sponges inserted, and normal postoperative radiographs were also used for validation. Image interpretations of the phantom radiographs achieved 100% sensitivity and specificity. The composite radiographs’ sensitivity was 97.7%, while the specificity was 83.8%. Normal postoperative radiographs used to determine false positive rates achieved a specificity of 86.6%, while in cadaveric radiographs, both specificity and sensitivity of more than 90% were realized. More advanced technologies are required to incorporate the solution into existing hardware.

### Prevention

Accidental retention of surgical sponges is easily preventable through the implementation of standardized counting procedures, but it persists due to human errors [33]. Surgical gauzes are small and similar to human tissues when soaked in blood, making it difficult to detect them when retained. In [27], the authors proposed an image processing system that uses the video captured by the endoscope during laparoscopy operations to track the gauze. Using texture analysis techniques, the application divides the video into different frames that are analyzed to determine the presence of a gauze pattern. Testing of the algorithm was performed using clean gauze, some that were slightly stained and others that were soaked in blood. To achieve reliable results, convolutional neural networks (CNN) and local binary patterns (LBP) were used. The LBP algorithm achieved sufficient results (98% precision and 94% sensitivity) for robust detection even when the gauzes are soaked or stained. Superior outcomes are obtained with the CNN algorithm, achieving 100% precision and 97% sensitivity. However, real-time detection using standard hardware is unattainable due to high computational requirements.

In minimally invasive surgery (MIS), tracking surgical tools in real-time has various uses for computer-assisted interventions (CAIs). Visually tracking approaches are meant to track surgical instruments in real time [34]. However, several methods have proved ineffective due to motion blur, poor illumination, specular reflections, shadows, and occlusions. In [28], a spatial transformer network (STN) and spatiotemporal context (STC) were used to propose an automated real-time technique for 2D tool detection and tracking. Their solution merged CNN with specially trained STN and STC to recognize the tool quickly and reliably. They used eight pre-existing online and internal datasets to compare their approach to four other generic CAIs’ visual tracking techniques, including in vivo abdominal, cardiac, and retinal clinical settings using diverse surgical equipment. The investigations found that the method’s accuracy and speed were both outstanding. Even in the most challenging environments, it could track a surgical tool in real-time without labeling with accuracy comparable to, and sometimes better than, most cutting-edge tracking systems. The technique would need to be refined further, focusing on multi-instruments and occlusion scenarios.

In [35], the study aimed to create an algorithm for detecting surgical equipment’s distal end by employing object identification and DL. Nine video recordings of carotid endarterectomies were used for training and testing purposes. As annotated data, regions of interest (ROIs) of 32 × 32 pixels were produced and positioned at the distal end of the surgical tool within the video frames. These ROIs were subjected to data augmentation techniques. The model for training was a CNN based on YOLOv2. The researchers evaluated the detectors to determine the average detection precision. The YOLOv2 model predicted the central coordinates of bounding boxes used in this study’s approach. The detection rate was calculated using the test dataset. In the absence of data augmentation, the mean accuracy for the ROIs was 0.4272 ± 0.108. The mean accuracy with data augmentation, measured 0.7718 ± 0.0824, on the other hand, demonstrated a considerable improvement above the scenario without data augmentation. The detection rates were 0.6100 ± 0.1014 and 0.9653 ± 0.0177, respectively, when the computed coordinates of center points within 8 × 8 and 16 × 16 were included. The researchers predicted that the proposed method would be effective in analyzing surgical records.

Real-time monitoring of surgical procedures could enhance the safety of surgical procedures and prevent instances of retained sponges. In [36], a laparoscopic video was utilized to obtain 4003 hand-labeled frames to be used as a segmentation dataset. Several baselines were analyzed to prove the dataset potential: detection with YOLOv3, segmentation using U-Net, and coarse segmentation. Although YOLOv3 was appropriate for real-time execution, it provided a modest recall. On the other hand, coarse segmentation lacked inference speed. At the same time, U-Net provided a satisfactory speed-quality compromise, running above 30 frames per second (FPS) with an intersection over union (IoU) of 0.85. The good compromise the U-Net achieves is evidence that performing precise and real-time gauze segmentation is possible.

The authors in [29] developed a novel hierarchically organized dataset for research emphasizing intelligent surgical equipment management. A four-level hierarchical framework and 360 separate surgical instrument categories were applied to the dataset. This structure was made up of twelve packs, 35 sets, and two specials. The researchers used CNNs in various approaches to evaluate this dataset’s picture categorization and retrieval abilities. These approaches involved using several CNN training strategies, including adding past knowledge using a taxonomic hierarchy tree structure. The team investigated how image size and the number of photos in each class influenced the models’ ability to forecast. An in-depth study was conducted to map image characteristics and class embeddings within a semantic space using semantic similarity scores across categories. Surprisingly, these experiments revealed that adding prior knowledge considerably improved the performance of picture retrieval on the dataset.

Keeping a record of surgical equipment is critical to ensuring surgical safety and patient welfare. However, due to the inherent uncertainties in manual record-keeping methods, instrument omissions or miscounts are possible [37]. Using computer vision technology makes the instrument counting technique more efficient, which also helps eliminate medical conflicts while developing medical information technology. Despite this, there are complications during the counting procedure. Surgical tools may be tightly packed or mutually obstructive, and their ability to be recognized may be influenced by lighting circumstances.

Furthermore, the presence of similar instruments with minor changes in appearance and shape makes identification more challenging. To overcome these challenges, the researchers in [38] updated the YOLOv7x object detection algorithm for surgical equipment detection. First, the RepLK Block module was introduced to the YOLOv7x backbone network, increasing the effective receptive field and boosting the network’s capacity to learn from features. Second, the neck module of the network was upgraded to add the ODConv structure, which significantly improved CNN’s ability to extract features while capturing richer contextual data. To simplify model training and evaluation, the researchers compiled the OSI26 dataset, which includes 452 pictures of 26 surgical equipment. Empirical results demonstrated the updated algorithm’s superior accuracy and durability in surgical equipment detection tasks. For the F1, AP, AP50, and AP75 parameters, the baseline was exceeded by 4.6%, 3.1%, 3.6%, and 3.9%, respectively, with 94.7%, 91.5%, 99.1%, and 98.2%. The proposed method has substantial advantages compared to other top object detection algorithms. These findings proved the method’s ability to detect surgical tools accurately, improving surgical safety and protecting patient health.

Table 4 includes the imaging modalities, metrics, key findings, and the main advantages and disadvantages of the DL methods described in [27–32, 35, 36, 38].

**Table 4:** Deep learning methods key findings, prominent advantages, and disadvantages

Modality (Dataset)	Metrics	Key findings	Advantages	Disadvantages
Optical imaging (various surgical tools​) [28], 2019	• Accuracy: 100%	• The paper compares the proposed method with four others. • Uses eight diverse surgical video datasets. • It covers varied surgical scenarios and conditions. • Demonstrates strong accuracy and speed in experiments. • Tracks tools label-free in real-time, excelling in tough cases.	• The method tracks surgical tools in real-time without labels, saving time. • Enhances accuracy and speed of surgical tool tracking. • Achieves high performance in accuracy and speed, improving procedures.•	• Method lacks real-time object tracking, limiting specific procedures. • Not effective with occlusion and multi-instruments, impacting accuracy. • Experiments on limited datasets may hinder broad usability. • High computational time (2.5 s) hampers real-time use on i3 CPU.
Optical imaging (4891 background, 1782 gauze) [27], 2020	• Precision: 100% • Sensitivity: 97%	• Paper presents an image processing system for gauze tracking in endoscope video. • Results based on the average of 110 simulated laparoscopic images in different conditions.	• The method achieves a highly accurate and sensitive real-time detection of gauzes. • The program analyzes the footage invisibly and without human intervention. • The algorithm is capable of tracking gauzes in real time. • The system is convenient and straightforward to use.	• The high computational demand of the CNN approach prevents real-time processing with standard hardware. • Limited testing in a laparoscopic simulator; more testing in real surgical scenarios is required. • The algorithm may not detect gauze hidden or obscured by tissues/organs in the video.
Optical imaging (4608 images) [35], 2020	• Detection rates: 0.6100 ± 0.1014 and 0.9653 ± 0.0177. • Average precision (AP) without augmentation: 0.4272 ± 0.108. • AP with augmentation: 0.7718 ± 0.0824	• The Mann-Whitney U-test was used to compare the impact of augmentation. • Evaluated detection with AP, LAMR, and FPS. • MATLAB’s Computer Vision Toolbox assessed bounding boxes.	• The algorithm enables real-time surgery monitoring. • Data augmentation notably enhances the algorithm’s accuracy and reliability. • Offers innovative surgical tool detection approach, enhancing outcomes and safety. • Contributes to expanding research on DL’s role in surgery advancement.	• Limited sample size (nine carotid endarterectomy videos) hampers generalization. • The study focused only on distal end detection, overlooking tissue factors. • Lack of real-time evaluation for practical surgery application. • Absence of comparison with existing detection methods. • Ethical and legal concerns, like patient privacy and liability, are unaddressed.
Optical imaging (4003 frames) [36], 2022	• Precision: 94.34% • Recall: 76.00% • F1 score: 84.18%	• Paper presents quantitative and qualitative results on the proposed dataset. • YOLOv3 offers real-time execution but modest recall. • Coarse segmentation is satisfactory but slow.	• U-Net achieves real-time segmentation balance. • Gauze detection automates surgical tasks and aids in video analysis. • Datasets and baselines offer a foundation for future research in the field.	• The dataset is limited to specific gauze types and surgery, not covering all scenarios. • Evaluation metrics include recall, MCC, and AP, possibly inadequate. • There is no comparison with existing methods in the field. • Lack of clinical validation impacts real-world applicability.
X-ray imaging (5085 images) [30], 2022	• Sensitivity: 85% • Specificity: 73% • AUC: 0.87	• DL is better for lung regions (AUC and sensitivity), and heart and subdiaphragmatic regions are equally high-performing (AUC > 0.9). • DL had lower specificity for all regions except the heart than human observers.	• The model has higher sensitivity, indicating a superior ability to identify surgical sponges in images. • The DL model enhances human visual examination while reducing the number of surgical items that are overlooked and not retained. • Improved model’s accuracy and reliability • Combining X-ray images from routine post-operative radiography and surgical sponges may allow for more precise detection of surgical sponges.	• Bias potential due to fusion with standard, not post-op, radiography. • Limited to chest sponge detection; further research is needed. • Medical staff’s familiarity with image interpretation could impact the study. • They relied on accessible NIH databases, affecting result generalizability. • There is no comparison with other RSI detection methods in the study.
Ultrasound imaging (7121 images) [31], 2022	• Accuracy: 99% • Sensitivity: 99% • Specificity: 99%	• Fully automated algorithm for detecting cotton balls in the brain. • It is integrated into the web app for rapid ultrasound image detection. • Actual in-human dataset use prevents foreign body retention. • Large, diverse ex vivo porcine brain ultrasound dataset for DL model.	• The method is non-invasive and safe; it uses ultrasound to detect cotton balls in the brain. • The automated algorithm integrates into the workflow and is time-efficient. • The algorithm achieves high accuracy, sensitivity, and specificity. • The first use of a real in-human dataset; prevents accidental retention, clinically relevant.	• Limited by a small sample (2 human brains), not generalizable. • Only cotton ball detection was studied; no assessment of other foreign bodies. • No real-time surgery evaluation; limits clinical use insight. • There is no comparison with human experts’ performance. • Not tested for foreign bodies outside the brain; limited applicability to other surgeries.
Optical imaging (15,522 surgical tool images) [29], 2022	• Accuracy: 88% • F1-score: 86% • Top-5 accuracy: 100% • Hierarchical accuracy: 84%	• Good classification, limited hierarchy impact. • Embedding strategies aid retrieval. • Single image retrieval is robust, and embedding helps with similarity. • Semantic data boosts image retrieval. • Hierarchy and semantics enhance performance.	• CNN strategies enhance dataset structure. • Semantic info boosts content-based image retrieval quality. • Research findings applicable in surgical tool management. • Insight into organizing tools for efficient surgical procedures.	• Limited dataset focus may hinder generalization. • Single dataset experiments limit real-world applicability. • Lack of detailed computational resource analysis. • There is no comparison with the current top methods for image tasks.
Optical imaging (452 images) [38], 2023	• Precision: 92.6% • Recall: 97.0% • FI-score: 94.7% • AP: 91.5%	• Paper’s algorithm enhances YOLOv7x for surgical instrument detection. • The proposed method shows higher accuracy and robustness. • Ablation experiments validate proposed improvements for instrument detection.	• Improved YOLOv7x algorithm enhances surgical instrument detection. • RepLK Block and ODConv improve the receptive field shape learning and context. • The approach accounts for obstacles such as closely packed equipment, occlusions, and varying lighting conditions, making it suitable for actual surgical circumstances.	• Errors are likely for similar-looking instruments. • Possible errors for similar-looking instruments. • The method works for standard OR instruments; special ones need specific methods. • Lighting variations impact accuracy.
X-ray radiography (5024 images) [32], 2021	**Phantom** • Sensitivity: 100% • Specificity: 100% **Composite** • Sensitivity: 97.9% • Specificity: 83.8% **Cadaver** • Sensitivity: 97.7% • Specificity: 90.4%	• CAD software developed for preventing RSIs. • They are trained to detect surgical sponges. • High sensitivity and specificity in various radiographs. • CAD system has the potential to enhance patient safety.	• The CAD software enhances patient safety through the detection of RSIs. • Reduces human error, improving surgical sponge detection accuracy. • Achieves high sensitivity and specificity in detecting surgical sponges. • Potential for cost-effective, practical use without extra equipment.	• The software specializes in detecting specific surgical sponges, not all retained items. • Technology’s applicability to other surgical instruments might be limited. • The study used a small sample of radiographs, potentially lacking generalizability. • No evaluation of CAD in larger clinical settings. • There is no assessment of CAD’s impact on patient outcomes or costs.

## Datasets

The dataset used in [17] comprises 790 X-ray images, with a staggering 25,638 negative box locations for non-RSI candidates and 561 positive box places for RSIs such as sponges. This semi-automatically labeled dataset is critical for teaching the automated identification system to distinguish between RSIs and non-RSI regions and aiding the training and testing of the system. Furthermore, the author’s ongoing efforts to add synthesis data to the dataset reflect their commitment to expanding its scope, ultimately improving the accuracy of RSI recognition in radiological images.

The authors of [19] emphasize the significance of using multiple datasets to advance various medical and surgical imaging aspects: they used three types of datasets in their study. The “NeuroSurgicalTools Dataset” contains 2476 monocular images ranging in size from 612 × 460 to 1920 × 1080 pixels captured during actual neurosurgery procedures. It is divided into training (1221 images) and testing (1255 images) halves as an essential tool for building and assessing algorithms for neurosurgical image processing. The “EndoVisSub Dataset,” a MICCAI 2015 endoscopic vision challenge component, comprises two sub-datasets, one robotic and one non-robotic. The robotic sub-dataset contains videos for training and testing at a resolution of 720 × 576 pixels. The non-robotic sub-dataset includes 160 in vivo abdominal images for training. Despite lacking extensive dataset metadata, the “FetalFlexTool Dataset” focuses on images of a flexible McKibben actuator used in fetal surgery. Collectively, these datasets offer specialized imaging data for medical and surgical research, covering neurosurgery, endoscopy, and fetal surgery tool analysis. These datasets are diverse, allowing the authors to investigate and build algorithms that aided in solving the issue of retained surgical artifacts in various surgical procedures and environments.

In [27], a dataset of images from laparoscopic recordings was employed, with 4891 blocks labeled as a backdrop and 1782 as gauze. These graphic blocks depicted various gauze conditions, including clean, unclean, and damp. In their study, 75 blocks were used for analysis, specifically to develop a pattern histogram, while 25 blocks were used for testing, which included the construction of receiver operating characteristic (ROC) curves. The authors used a dataset of image blocks from laparoscopic recordings to examine several LBP variants. They used the area under the curve of the receiver operating characteristic (AUC-ROC) to quantify their performance. The study also includes a clean gauze video with a Storz Telecam endoscope in a laparoscopic simulator. This video was used during the experimental setup and testing phases. The study examines how successfully LBP variations distinguish between gauze and non-gauze regions using image data, ROC curves, and AUC-ROC computations, which is significant for developing tools to avoid complications from RSIs.

In [36], the dataset contains 42 video files, 30 of which have RSIs and 12 of which do not. There are 33 min of RSI videos and 13 min of non-RSI videos. It was shot with a PAL color STORZ TELECAM one-chip camera head with a 752 × 582-pixel image sensor. However, it is based on simulated scenarios using animal organs rather than actual treatments. This dataset is extremely useful for RSI research since it provides a wide range of operating settings, such as varied gauze conditions and tool presence, which are required for developing and analyzing RSI detection algorithms.

In [28], the authors employed eight distinct datasets sourced internally and via the Internet to test a novel method for real-time monitoring of surgical equipment during MIS. In vivo, abdominal, heart, and retinal surgeries, as well as various clinical settings and surgical instruments, were all included in these datasets. The dataset is critical for evaluating how well the suggested tracking system may minimize the potentially fatal retention of surgical equipment since it replicates difficult situations seen in MIS. They addressed various standard and challenging conditions, including motion blur, low lighting, reflections, shadows, and obstructions. The study compared the precision and real-time performance of four existing generic CAI tracking methods to the suggested tracking approach, which was measured in FPS. This dataset’s test video footage, which includes embedded ground truth data, was utilized to evaluate tracking algorithms.

In [35], specialized software was developed to select the 32 × 32-pixel (ROIs on surgical equipment and collect object names and ROI coordinates. This resulted in a thoroughly vetted dataset, which the researchers used. The dataset was divided into nine subgroups for nested cross-validation to guarantee that patient images were separated between training and testing. The training dataset, which included 4096 training images obtained from eight patients and 512 for testing, performed better after data augmentation with image rotation. The separation of patient images in training and testing is ensured by nested cross-validation with nine subgroups, increasing the model’s robustness for identifying RSIs in various settings.

The HOS-PITools dataset in [29] contains 360 surgical instrument classifications organized hierarchically across four tiers, which is an essential resource for the study. CNNs and other DL approaches can be investigated for usage in image categorization and retrieval applications. The study analyzes the effects of image size and image count per class on model performance to better understand the semantic links among surgical equipment. It also explores mapping image properties and class embeddings in a semantic space. This dataset greatly promotes research on RSIs in the surgical domain by providing a comprehensive and hierarchically organized model evaluation and development resource.

In [30], the study used two crucial datasets for DL, each with its collection of numerical features. The initial dataset had 8619 chest radiography images from 51 patients, which were obtained utilizing precise imaging parameters: a 200 cm source-to-detector distance, 120 kVp tube voltage, and 160 mA tube current. The second dataset included surgical sponge pictures obtained by imaging three sponges under controlled settings using a 100 cm source-to-detector distance, 66 kVp tube voltage, and 1.6 mA tube current-time product. These datasets are critical for detecting retained surgical objects, contributing significantly to patient safety during surgical procedures.

In [31], the dataset contains photographs of pig brains labeled with cotton’s location in the brain. The images were divided into three groups based on their diameter: training (70%), validation (15%), and test (15%). The edges of these photos were enhanced with anisotropic diffusion, and the pixel intensity was smoothed. They were also scaled and modified to preserve the values between 0 and 1. In addition, lower-resolution human neurosurgical pictures were handled using contrast-limited adaptive histogram equalization (CLAHE), which improves contrast. This dataset’s thorough curation and range of image types are critical for developing and testing RSI detection algorithms, which will considerably improve surgical precision and patient safety.

The OSI26 dataset used in [38], containing 452 images and 26 surgical instruments, is essential in exploring surgical tool detection for reducing RSIs. Each image was annotated and standardized at 1980 × 1080 pixels. Several data augmentation approaches, such as translation, scaling, flipping, brightness, hue, saturation adjustments, and mixup and mosaic methods, were applied to boost model performance. The data was divided into training, validation, and testing sets (in an 8:1:1 ratio) to guarantee category balance. Because of its broad diversity, well-annotated images, and additional data, the usefulness of this dataset rests in developing an improved algorithm for surgical instrument detection, which is critical for minimizing the incidence of RSIs during surgery.

The dataset in [32] included a validation set of 470 images to evaluate the program’s effectiveness and a training set of 4,554 composite radiographs to train the CAD algorithm. Various radiograph types were included in the validation set, including 1,776 normal postoperative radiographs and 369 cadaver radiographs. The software’s capacity to find surgical sponges inside the body was tested in several conditions, including controlled phantoms and cadaver radiography. The study’s findings revealed that the CAD software is susceptible to detecting retained surgical sponges, significantly increasing patient safety and medical imaging. DL was used to train the software, which was fed a dataset of composite radiographs that combined images of surgical sponges with usual postoperative X-rays. Various radiograph types, including cadaver, composite, phantom, and standard postoperative images, were used to properly examine the software’s efficiency. An upgraded model based on Faster R-CNN was used to improve object detection. The accuracy was evaluated using sensitivity, specificity, and precision rate criteria. One critical feature that qualifies the DL model for clinical use is its ability to transfer information across multiple radiological categories. Essentially, this technique demonstrates how DL may improve the detection of retained surgical sponges, enhancing patient safety in medical imaging.

In [20], the research used radiographic images from 10 cadavers to develop and test a CAD system for detecting radiopaque, ultimately eliminating inadvertent surgical equipment retention. The dataset used in this study contains 700 radiographs, 410 of which include radiopaque features randomly placed, and the remaining 290 do not. The cadavers were positioned in several orientations and rotations to properly mimic various clinical situations, resulting in a dataset that accurately reflects real-world environmental variables. Objects commonly seen in intraoperative radiographs were deliberately placed in the cadavers to mimic surgical circumstances.

The dataset employed in [18] comprises radiographic images of cadavers with surgically implanted needles in their chest and abdomen regions, replicating instances in which RSIs appear in medical imaging. The collection had 108 radiographs with 116 needles embedded at various places from 19 different cadavers. The dataset was separated into a training subset of 53 radiographs with 59 needles and a testing subset of 55 radiographs with 57 needles to facilitate model training and evaluation. The testing subset also included 100 radiographs of cadavers without needles to assess the CAD system’s false-positive rate.

## Discussion

It is vital to highlight that the use of ML and DL in the diagnosis and prevention of RSIs could result in increased surgical safety and improved diagnosis accuracy. Each technology has its own set of advantages and disadvantages in this critical medical field, as demonstrated in the reviewed articles. To fully comprehend the impact, it is crucial to examine their characteristics, benefits, and drawbacks before presenting a comparison.

### Strengths and limitations

ML is applied in predictive analysis, utilizing various risk factors to anticipate RSIs. It aids in real-time monitoring of surgical instruments and sponges and enhances the accuracy of postoperative scans. ML could enable the creation of training simulations, the detection of dangerous surgical workflow patterns, the automation of inventory management, and the interface with EHR for thorough documentation. ML is adaptable to any data, and its predicting capabilities in identifying RSIs may be integrated into current systems such as EHR.

DL is well-known for its superiority in advanced image recognition, which is required for real-time tracking of surgical items. DL is also important in constructing customizable training and could be connected to EHR for more comprehensive database assessment. DL demonstrates outstanding performance capabilities in pattern identification, particularly in image and video analysis. It does, however, offer better accuracy RSI scanning with advanced analytics for complex datasets.

Despite these strengths, both technologies have limitations. ML performance is largely dependent on data quality and quantity, yet it can struggle with intricate pattern recognition. It also carries the risk of overfitting. DL is powerful, but the cost of operation is expensive, and this operation is similar to a black box since its method of operation is opaque or unclear. It is a significant challenge because it requires a huge, well-labeled dataset during the training process.

### Comparative analysis

ML is adaptable and can be used at any surgical stage to provide precise predictions in a high-risk setting. DL is better positioned in applications that require precision, such as real-time monitoring and post-operative scan interpretation, because of its high-quality visual analysis. However, the complexity and resource demands of DL can limit its accessibility, particularly in resource-constrained contexts. ML is significantly easier to employ because it uses less complex and less expensive software and hardware and requires less data.

Overall, ML and DL could be useful in detecting and diagnosing RSIs, each with its unique set of strengths. The decision between them should be based on the nature of the task, the availability of resources, and the desired output. An ideal approach might involve leveraging the strengths of the two techniques to provide a holistic solution to enhance surgical safety and patient care.

## Future directions on RSI occurrence minimization

### Data integration and connectivity

Reducing the occurrence of RSIs requires the use of cutting-edge technologies. This undertaking requires connectivity and data integration. Centralized data platforms can be built up to aggregate data from various sources in the surgical setting, such as inventory management systems, electronic health records (EHR), and surgical team communications. This complete integration provides a comprehensive view of the surgical operation, allowing for real-time monitoring and analysis. Furthermore, the integration of Medical Internet of Things (MIoT) devices creates a connected network that offers real-time information about surgical items.

### Blockchain technology

Blockchain technology can significantly minimize RSI incidence. By exploiting its inherent properties of transparency and unchanging recording, it is possible to ensure unchangeable documentation of each stage of the surgical operation. Some parts of the counting process can be automated using smart contracts, which are programmable blockchain components. Smart contracts, for example, can send out alerts or alarms if variations in the surgical item count are discovered.

### Computer vision and image recognition

The combination of image recognition and computer vision technology holds a lot of promise for RSI prevention. Automated count verification is possible with computer vision systems that analyze visual data in real-time and cross-reference it with pre-surgical checklists to ensure all objects are appropriately retrieved. Identifying and classifying sponges and surgical instruments can be taught to object recognition algorithms, which will build a visual database for cross-referencing and validation while performing surgery.

### Augmented reality

Augmented Reality (AR) stands out as a transformative technology in the context of RSI prevention. AR overlays can provide real-time visual cues to surgical teams regarding the location and status of instruments. For example, AR can highlight missing items or offer a digital checklist that updates dynamically as instruments are used and accounted for. Moreover, AR can be employed for remote expert assistance during surgeries, allowing experts to view the surgical field and provide guidance, including double-checking instrument counts through a virtual interface.

## Conclusion

RSIs, objects inadvertently left within patients’ post-surgery, pose significant risks to patient safety, healthcare professionals, and institutions. Per 1,000 abdominal surgeries, there are approximately 0.3 to 1.0 occurrences of retained surgical objects [2]. Patients in such situations face serious consequences, such as infections, organ damage, prolonged hospitalization, and, in some cases, fatal outcomes. Given the limitations of current RSI prevention methods like radiography and manual counting, there is growing interest in advanced solutions such as ML and DL. Although research on these advanced solutions has produced promising results, the potential of this technology requires more exploration. This paper underscores the significance of integrating multiple RSI prevention and diagnosis approaches into a comprehensive strategy for optimal results. The evaluation explores cutting-edge medical technology improvements. It investigates the potential for proactive diagnosis and prevention of RSIs using DL and ML. DL and ML algorithms can efficiently analyze vast amounts of data to identify patterns related to RSIs, reducing the need for extensive human intervention, and enhancing precision. Once trained on diverse datasets, these models offer highly accurate RSI prevention, ultimately improving surgical outcomes and patient safety.

Leveraging advanced technology represents a promising strategy to mitigate the occurrence of RSIs, a critical concern in healthcare. Future directions in this field encompass developing real-time intraoperative monitoring systems, refining AI models to enhance accuracy, and exploring cutting-edge technologies to assist surgical teams in preventing RSIs. As technology continues to advance, there is a substantial potential to significantly reduce the occurrence of RSIs, enhancing patient safety and elevating the standard of surgical care. The fusion of emerging technologies with the current ones such as ML and DL presents an innovative solution to the pressing issue of RSIs, illustrating the healthcare industry’s commitment to using innovation to create safer, more efficient, and patient-centric surgical environments.

## Data Availability

Data sharing does not apply to this article as no datasets were generated or analyzed during the current study.

## Abbreviations

AUC: ROC Area under the curve of the receiver operating characteristic
CAD Computer: aided detection
CAIs Computer: assisted interventions
CLAHE Contrast: limited adaptive histogram equalization
CNN: Convolutional neural networks
EHRs: Electronic health records
FCN: Fully convolutional networks
FPS: Frames per second
IoT: Internet of things
IoU: Intersection over union
LBP: Local binary patterns
LD: Linear discriminant analysis
MIoT: Medical internet of things
MIS: Minimally invasive surgery
OR: Operating room
PACS: Picture archiving and communication system
R-CNN: Region-based convolutional neural network
RFID: Radio-frequency identification
RNNs: Recurrent neural networks
ROC: Receiver operating characteristic
ROIs: Regions of interest
RPN: Region proposal network
STC: Spatiotemporal context
RSIs: Retained surgical items
STN: Spatial Transformer network
